# In-Situ Synthesis of Hydrogen Titanate Nanotube/Graphene Composites with a Chemically Bonded Interface and Enhanced Visible Photocatalytic Activity

**DOI:** 10.3390/nano8040229

**Published:** 2018-04-08

**Authors:** Juan Yang, Jun You, Jun Dai, Yumei Chen, Yao Li

**Affiliations:** 1 Institute of Chemical Safety, School of Safety Science and Engineering, Henan Polytechnic University, Jiaozuo 454003, China; youjunhpu@163.com (J.Y.); daijun@hpu.edu.cn (J.D.); leayao35@hpu.edu.cn (Y.L.); 2 Institute of Applied Chemistry, College of Chemistry and Chemical Engineering, Henan Polytechnic University, Jiaozuo 454003, China

**Keywords:** photocatalytic selective oxidation, Ti–C bonds, hydrogen titanate, benzylic alcohols, interfacial charge transfer

## Abstract

Hydrogen titanate nanotube (HTT)/graphene nanocomposites are synthesized by hydrothermal reduction of graphene oxide (GO) and simultaneous preparation of nanotubular HTT via an alkaline hydrothermal process. By using this facile in-situ compositing strategy, HTT are densely supported upon the surface of graphene sheets with close interface contacts. The as-prepared HTT/graphene nanocomposites possess significantly enhanced visible light catalytic activity for the partial oxidation of benzylic alcohols. The amount of graphene has significant influence on catalytic activity and the optimal content of graphene is 1.0 wt %, giving a normalized rate constant *k* of 1.71 × 10^−3^ g/m^2^·h, which exceeds that of pure HTT and HTT/graphene-1.0% mixed by a factor of 7.1 or 5.2. Other than the general role of graphene as a high-performance electron acceptor or transporter, the observed enhancement in photocatalytic activity over HTT/graphene can be ascribed to the improved interfacial charge migration from enhanced chemical bonding (Ti–C bonds) during the in-situ compositing process. The formation of Ti–C bonds is confirmed by XPS analysis and the resulting enhanced separation of photoinduced charge carriers is demonstrated by electrochemical impedance spectra and transient photocurrent response.

## 1. Introduction

In the last several decades, the photocatalytic process in semiconductors has been attracting more and more attention as an advanced green technology for contaminant purification, energy production, and selective organic synthesis [1,2,3]. The effectiveness of photocatalysis is primarily dependent on three aspects: optical absorption, charge separation, and catalytic reaction upon the surface of the semiconductor catalyst. Further research shows only a small part of photoinduced charges can migrate successfully to the surface of catalysts and initiate catalytic reactions. As a result, the efficient charge transfer on the surface of a semiconductor photocatalyst is widely considered to be very critical, being an elementary process related to photocatalytic application.

Among various photocatalysts, TiO_2_-derived nanosized materials appear to be a potential candidate, owing largely to their excellent performance, long term stability, low toxicity, and cost [4,5]. In the field of photocatalysis, hydrogen titanate (HT), especially nanotubular HT has been principally used to fabricate diverse TiO_2_ nanostructures, such as TiO_2_ nanotubes, nanowires, and nanofibers [6,7,8]. Their unique one-dimensional structural characteristics provide the preconditions necessary for suitable catalysts under ultraviolet or visible illumination [9,10]. For example, HT calcined at 300–500 °C, possessing TiO_2_(B) or anatase phase, exhibits higher catalytic performance for pollutant degradation than P25-TiO_2_ [11,12]. However, the inherently rapid recombination of photogenerated charge carriers within TiO_2_ or titanate remains problematic. Numerous strategies have been studied to tailor the photocatalytic process and promote catalytic performance. Particularly, fabrication of carbon-titanate heterojunction composites has been identified as one of the most feasible strategies to enhance the separation of electron-hole pairs [13,14,15].

Owing to the great surface area of graphene and its excellent electronic transport properties, many studies have been carried out on fabricating composite catalysts of graphene and semiconductor particles in an attempt to promote photocatalytic activity [16,17]. Under light illumination, it is found that photoinduced electrons migrate from photocatalysts to the surface of graphene and suppress the recombination of electron-hole pairs, thereby enhancing the oxidative or reductive reactivity of the photocatalytic process [18]. Further research demonstrated that the interfacial action between the semiconductor and graphene can notably influence the interfacial electron migration, which is thought to be a critical step of heterogeneous photocatalysis [19,20,21,22]. Zhang et al. have reported the synthesis of graphene-Bi_2_WO_6_ composites with strong covalent bonding between graphene and Bi_2_WO_6_ nanoparticles. The improved photocatalytic performance for H_2_ and O_2_ production were ascribed to the efficient electron migration and separation of photoinduced charge carriers through the chemically bonded interaction [20]. Xie et al. have recently reported the existence of a chemically bonded interface, via the formation of C–Ti bonds, between graphene sheets and TiO_2_ nanoparticles. This chemical bonding effect plays a pivotal role in the interfacial electron migration, which makes TiO_2_/graphene composites possess an outstanding catalytic activity for the photo-oxidation of formaldehyde, compared to a mechanically mixed sample [22].

The selective oxidation of alcohol to carbonyl compounds is a significant chemical conversion process. Generally, the partial oxidations of alcohols are implemented with hazardous or toxic stoichiometric oxidants under high temperature and pressure conditions [23]. Deteriorating environmental problems caused by chemical production processes have compelled us to seek green and economic alternative reaction systems. Recently, photocatalysis-based organic synthesis reactions have attracted wide concerns because they can be carried out using green oxidants (such as molecular oxygen) and solar energy under ambient temperature or pressure [3,24,25]. Several types of catalysts have been studied to achieve photocatalytic conversion of alcohols to the corresponding carbonyl compounds, including g-C_3_N_4_, HNb_3_O_8_ nanosheets, CdS, and TiO_2_-based composites [24,26,27,28]. What’s more, the selective oxidation of alcohols can be performed over TiO_2_ catalysts using visible light, although the photocatalysts themselves and the reactants can only absorb UV light [26,27]. In our previous report, hydrogen titanate (HT) nanotubes exhibited much higher visible light catalytic performance toward partial oxidation of benzylic alcohols, compared with HT nanosheets and nanofibers [28]. The superior photo-activity of HT nanotubes is primarily ascribed to their one-dimensional structural features, which include a high surface area, abundant unsaturated Ti sites, and efficient charge migration. Developing composite photocatalysts of HT nanotubes and graphene through an in-situ synthetic strategy can further improve catalytic performance for the selective oxidation of alcohols.

However, previously reported research on photocatalytic selective synthesis principally focused on reaction systems using TiO_2_ or TiO_2_ nanohybrids as photocatalysts [29,30,31]. The photocatalytic studies on titanate/graphene nanocomposites have largely focused on removing organic pollutants and splitting H_2_O for hydrogen production [32,33], whereas the selective chemical conversion using titanate/graphene composite photocatalysts has been scarcely researched. Furthermore, studies on the effects of chemical bonding on interfacial charge transfer and catalytic activity of titanate/graphene nanocomposites for photocatalytic partial oxidation of alcohols are also lacking.

In this paper, HT nanotube (HTT)/graphene nanohybrids with numerous heterogeneous interfaces are prepared via a facile hydrothermal method under basic conditions. Due to their one-dimensional structural characteristics, HT nanotubes can be uniformly coupled onto graphene to form close interfacial contacts. The presence of chemical Ti–C bonds between nanotubular HT and graphene are confirmed by XPS analysis. The HTT/graphene nanocomposites with chemically bonded interfaces exhibit exceptional photocatalytic reactivity toward the partial oxidation of benzylic alcohols under visible illumination. The influence of graphene content on the catalytic performance is investigated and a possible mechanism based on the experimental results is also proposed.

## 2. Materials and Methods

### 2.1. Materials

Graphite was purchased from Nanjing XFNANO Materials Tech Co., Ltd. (Nanjing, China). KMnO_4_, H_2_SO_4_, H_3_PO_4_, H_2_O_2_ (30%), NaOH, P25 TiO_2,_ and ethanol were obtained from Aladdin Industrial Inc. (Shanghai, China). Benzyl alcohol and its derivatives were purchased from J&K Scientific Ltd. (Beijing, China). 5,5-Dimethyl-1-pyrroline-N-oxide was obtained from TCI Development Ltd. (Shanghai, China). All the other chemicals were of analytical reagent grade and used as received without further purification. Deionized water was obtained using a Millipore Milli-Q system and used throughout the experiments.

### 2.2. Synthesis of HTT/Graphene Nanohybrids

To improve the hydrophilic and oxidation degree of graphite, a modified method was used to prepare graphite oxide [34]. In brief, a mixed solution of concentrated H_2_SO_4_/H_3_PO_4_ (volume ratio, 9:1) was added into a mixture of squama graphite and KMnO_4_ (weight ratio, 1:6). Then, the obtained mixture was heated for 12 h at 323 K under magnetic stirring. After being cooled down to room temperature, the mixture was poured into an icy solution of 30% H_2_O_2_. The solid was recycled by filtration, washed by deionized water, and vacuum-dried at 298 K for 12 h. The obtained graphite oxide (GO) was dispersed in water (0.5 mg/mL) and subjected to sonication for 60 min to form an aqueous dispersion of exfoliated GO.

In a typical synthesis process for HTT/graphene nanohybrids, 1.3 g of P25 TiO_2_ was added to 65 mL of concentrated NaOH solution (10.0 M) and was stirred for 30 min to form a uniform suspension. Then, a specific amount of GO dispersion was added to the above-obtained suspension under magnetic stirring. The weight ratios of GO to P25 TiO_2_ were 0, 0.2, 0.4, 1.0, 2.0, and 4.0%, and the as-prepared samples are labeled as HTT/graphene-x%, where x refers to the mentioned weight ratios. After further stirring for 90 min, the mixture was transferred into a 100 mL stainless steel autoclave and heated at 160 °C for 12 h. The obtained precipitate was separated by centrifugation, washed by deionized water, and re-dispersed for several cycles until a pH value of nearly 8 was obtained. The precipitate was finally washed with absolute ethanol and dried at 90 °C for 10 h to obtain HTT/graphene-x% nanocomposites.

For comparison, bare HTT and graphene samples were prepared using a similar process without GO or P25 TiO_2_ and NaOH solution. About 10 mg of graphene was added into 50 mL of deionized water and sonicated for 1 h. Then 1.0 g of as-made HTT was mixed into the graphene suspension under magnetic stirring. After being stirred for 2 h, the homogeneous suspension was treated by centrifugation. The precipitate was washed with deionized water and dried in a vacuum oven at 90 °C for 10 h. The as-obtained product is denoted as HTT/graphene-1.0%-mixed. For the preparation of the P25 TiO_2_/graphene-1.0% composite, 1.0 g of P25 TiO_2_ was added to 65 mL of deionized water and stirred for 30 min. 10 mL of GO dispersion (1.0 mg/mL) was added into P25 TiO_2_ suspension under magnetic stirring. After further stirring for 90 min, the mixture was transferred into a 100 mL Teflon-lined autoclave and heated at 160 °C for 12 h. The obtained precipitate was washed with deionized water and dried at 90 °C overnight. The as-synthesized sample is named as P25 TiO_2_/graphene-1.0%.

### 2.3. Characterization

X-ray diffraction measurements were conducted using a D8 Advance diffractometer (Bruker) with Cu Kα radiation (λ = 0.15418 nm). Raman spectra were recorded on a Labram HR 800 Raman spectrometer (Horiba Jobin Yvon, Paris, France) using 532 nm excitation light at room temperature. The morphological features of HTT/graphene nanohybrids were observed by a transmission electron microscope (TEM, Tecnai G20, FEI, Hillsboro, WA, USA) operating at 200 kV. FTIR spectra were acquired with a Nicolet 750 FTIR spectrometer in the range 400–4000 cm^−1^. X-ray photoelectron spectra were recorded using a Thermo Scientific ECALAB 250xi system with a monochromated Al Ka X-ray source. UV-visible diffuse reflectance spectra were collected on a UV-vis spectrophotometer (UV-2550, Shimadzu, Kyoto, Japan) using BaSO_4_ as the reflectance standard. The specific surface area of as-synthesized samples were measured by N_2_ adsorption-desorption with a Micrometrics ASAP 2020 apparatus.

Photoelectrochemical experiments were performed in a standard three-electrode system. Platinum foil and Ag/AgCl were used as the counter and reference electrodes, respectively. The working electrode was prepared by the following method: 40 mg of powder sample was dispersed in 0.2 mL of polyethylene glycol solution and ultrasonicated for 45 min to obtain a uniform slurry. The resulting slurry was then spread on a fluorine doped tin oxide (FTO) conducting glass and was dried at 90 °C to get HTT/graphene/FTO electrodes. The photocurrent was recorded at a bias voltage of 0.6 V on an electrochemical workstation (CHI-660C, Shanghai, China). The electrolyte was 0.1 M LiClO_4_ and nitrogen gas was continuously purged through the electrolyte solution. After being immersed in a benzyl alcohol solution for 1.5 h, the working electrode was irradiated using a 300 W Xe lamp with a 400 nm cut-off filter. Electrochemical impedance spectra (EIS) were measured using the above-mentioned three-electrode cell at 0.0 V with 0.1M Na_2_SO_4_ aqueous solution. Electron spin resonance (ESR) signals of the radicals trapped by DMPO were collected on a Bruker ER200-SRC spectrometer at ambient temperature.

### 2.4. Photocatalytic Experiment of Alcohol Oxidation

The partial conversion of benzyl alcohol was performed in a 20 mL round-bottom quartz glass flask. Benzyl alcohol (0.25 mmol) and the photocatalyst (50 mg) were dispersed into 5 mL of acetonitrile and the obtained mixture was bubbled with O_2_ for 30 min. This suspension was stirred magnetically for another 60 min to reach the adsorption-desorption equilibrium of the reactant alcohols and catalysts. The mixture was then irradiated using a 300 W Xe lamp with 400 nm cut-off filter under ambient temperature. At certain time intervals, a 0.5 mL mixture was withdrawn and filtered using a Millipore filter (pore size 0.45 μm) to remove the catalyst particles. The concentration changes of reactants in the remaining filtrate were quantitatively analyzed on a gas chromatograph (Agilent 7890) equipped with a standard FID detector and a DM-5 amine capillary column. Oxidation products were identified by comparing the retention times with the standards and further confirmed with GC-MS.

### 2.5. Radical Scavenging Experiments

In order to elucidate the photocatalytic mechanism, control experiments using different radical scavengers (*tert*-butyl alcohol, triethanolamine, and benzoquinone as the scavenger for the hydroxyl, photogenerated hole, and superoxide radicals, respectively) were performed via an analogous process of benzyl alcohol photo-oxidation, except that the radical scavenger (0.2 mmol) was included. The primary role of the active species were revealed by analyzing the changes of the benzyl alcohol conversion yield.

## 3. Results and Discussion

### 3.1. XRD, FTIR and Raman Spectra

**Figure 1:**
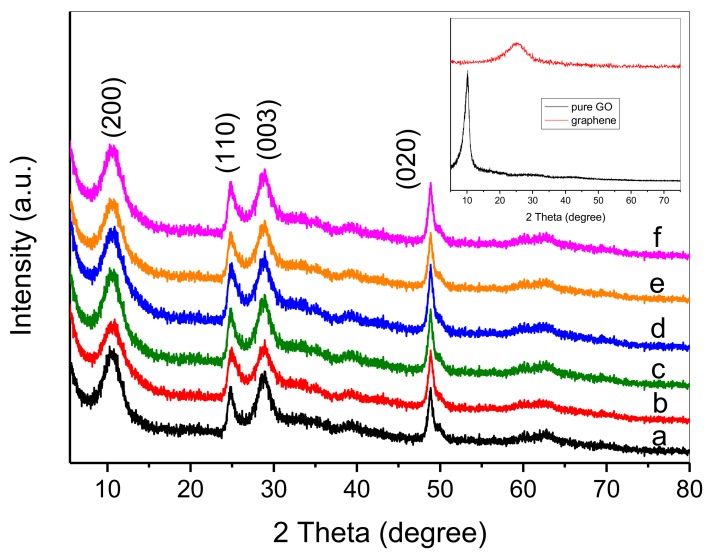
XRD patterns of hydrogen titanate nanotube (HTT)/graphene nanocomposites: (a) pure HTT, (b) HTT/graphene-0.2%, (c) HTT/graphene-0.4%, (d) HTT/graphene-1.0%, (e) HTT/graphene-2.0%, (f) HTT/graphene-4.0%. Inset: XRD patterns of pure GO and graphene.

Figure 1 shows the XRD patterns of as-synthesized samples including blank HTT and nanocomposites of HTT/graphene with different contents of graphene. Broad diffraction peaks are observed at 2θ = 10.7°, 24.7°, 29.0°, and 48.5°, which can be assigned to the characteristic crystalline phase peaks of layered hydrogen titanate [13,35]. For the sample of pure GO (inset of Figure 1), the diffraction peak at 2θ = 10.5° corresponds to the (002) reflection of stacked GO sheets. Additionally, pure reduced GO obtained in the absence of HTT indicates a broad (002) graphene reflection at 26.5°, which clearly demonstrates the conversion of GO to graphene [36]. However, in comparison with the XRD patterns of GO and graphene (inset of Figure 1), no diffraction peaks belonging to GO or graphene are observed in the XRD spectra of HTT/graphene nanocomposites. It is hard to distinguish the characteristic peak at 2θ = 10.5° of pure GO and the broad peak at 2θ = 10.7° assigned to (200) crystallographic planes of hydrogen titanate. The absence of these peaks might also be due to the low weight content and weak diffraction intensity of GO and graphene.

**Figure 2:**
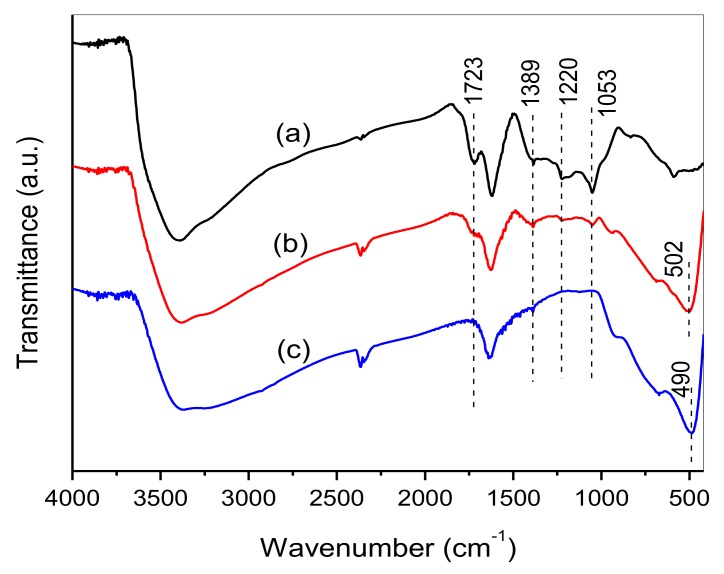
FTIR spectra of samples (a) GO, (b) HTT/graphene-1.0%, and (c) pure HTT.

The presence of graphene in the HTT/graphene nanocomposites could be confirmed using FTIR spectra (Figure 2). Bare GO exhibits several characteristic peaks belonging to oxygen functional groups, such as C=O stretching at 1723 cm^−1^, O–H deformation at 1389 cm^−1^, C–OH stretching at 1220 cm^−1^, and C–O stretching at 1053 cm^−1^ [20,34]. Compared with that of bare GO, the spectrum of HTT/graphene-1.0% shows a significant decline in intensity for peaks associated with oxygen-containing groups (i.e., C=O, O–H, C–OH, and C–O), indicating that GO in the composites is reduced. Additionally, the strong absorption band centered at 490 cm^−1^ can be assigned to the Ti–O–Ti stretching vibration in the pure HTT sample. A slight blue shift can be observed in the FTIR spectrum of HTT/graphene-1.0% (centered at 502 cm^−1^), suggesting an interfacial interaction between graphene and HTT in the nanocomposites.

**Figure 3:**
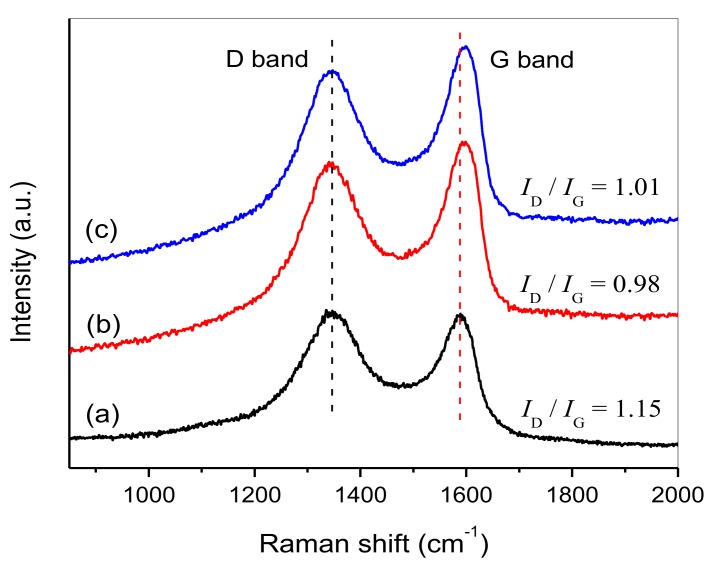
Raman spectra of (a) bare GO, (b) HTT/graphene-1.0%, and (c) HTT/graphene-4.0% composites.

Raman spectroscopy is also widely employed to characterize the electronic structure of carbon products. Two bands are observed in the 850–2000 cm^−1^ range for GO and HTT/graphene nanocomposites, as shown in Figure 3. They are the D band and G band of carbonaceous materials [36]. The former denotes a common characteristic of carbon sp^3^ defects, and the latter gives information about the in-plane vibrations of sp^2^ bonded carbon [21,34]. The intensity ratio between D band and G band (*I*_D_/*I*_G_) represents the relative concentration of defects in GO or graphene. Compared with bare GO, the calculated *I*_D_/*I*_G_ of the HTT/graphene composites are lower, demonstrating a lower concentration of defects in graphene. The decreased *I*_D_/*I*_G_ ratios in HTT/graphene nanohybrids imply the employed hydrothermal process facilitates the formation of more graphene without reducing the average size of sp^2^ domains. Furthermore, it is worth noting that a G band up-shift from 1588 to 1596 cm^−1^ is observed for HTT/graphene compared with GO and graphene, which gives evidence for chemical bonding of carbon materials [37,38]. The results indicate significant interaction between graphene and HTT, consistent with the results of the FTIR investigation.

### 3.2. TEM and Brunauer–Emmett–Teller (BET) Surface Area Analyses

**Figure 4:**
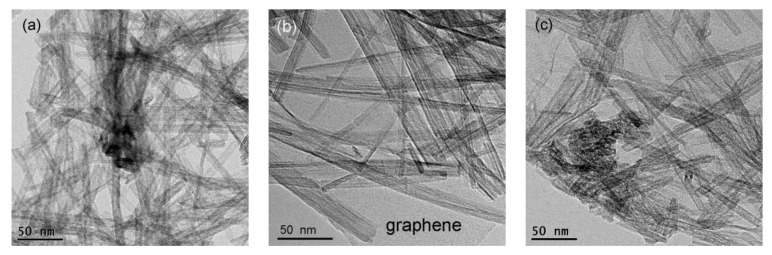
Typical TEM images of (**a**) pure HTT, (**b**) HTT/graphene-1.0%, and (**c**) HTT/graphene-1.0%-mixed nanocomposites.

The morphologies of HTT/graphene composites can be observed by TEM, as shown in Figure 4. Pure HTT exhibit a tubular framework structure, although, occasionally the aggregation of HTT can be seen (Figure 4a). Upon introducing GO, HTT are anchored onto the graphene sheets after hydrothermal treatment (Figure 4b). The surfaces of graphene sheets are covered by interconnected networks of HT nanotubes. The introduced GO acts as a substrate of the tightly packed HTT. It is noted from Figure 4b, that HTT occupy most of the available surface of graphene, leading to high loading amounts of HTT in as-synthesized nanocomposites. Compared to HTT/graphene-1.0% obtained via the in-situ synthesis strategy, the HTT/graphene-1.0%-mixed sample, prepared by the mechanically mixing of HTT and graphene, exhibits an inhomogeneous distribution of HTT on graphene, as shown in Figure 4c. These results demonstrate that the oxygen-containing functional groups of GO facilitate the nucleation and growth of HTT upon the graphene surface. It is beneficial for HTT to anchor directly on graphene sheets, consequently forming a uniform distribution and close interfacial contact between HTT and graphene.

N_2_ adsorption-desorption experiments were used to investigate the textual properties of the as-synthesized nanocomposites. The specific surface area, S_BET_ was calculated according to the classical Brunauer–Emmett–Teller (BET) theory and the corresponding data are summarized in Table 1. Table 1 indicates that all of the HTT/graphene composite samples possess larger S_BET_ than bare HTT, which is due to the huge surface area of graphene [20]. Additionally, the BET surface area of HTT/graphene hybrids increases with an increasing amount of graphene, reconfirming the influence of graphene in improving the total surface area of HTT/graphene samples. For instance, S_BET_ increases from 228.9 to 248.0 m^2^·g^−1^ when the content of graphene increases from 0.2% to 4.0% for as-synthesized HTT/graphene composites. There is no doubt that the enhanced S_BET_ can provide more surface active sites and facilitate the adsorption of the reactants, thus promoting heterogeneous catalytic reactions.

### 3.3. XPS Analysis

**Figure 5:**
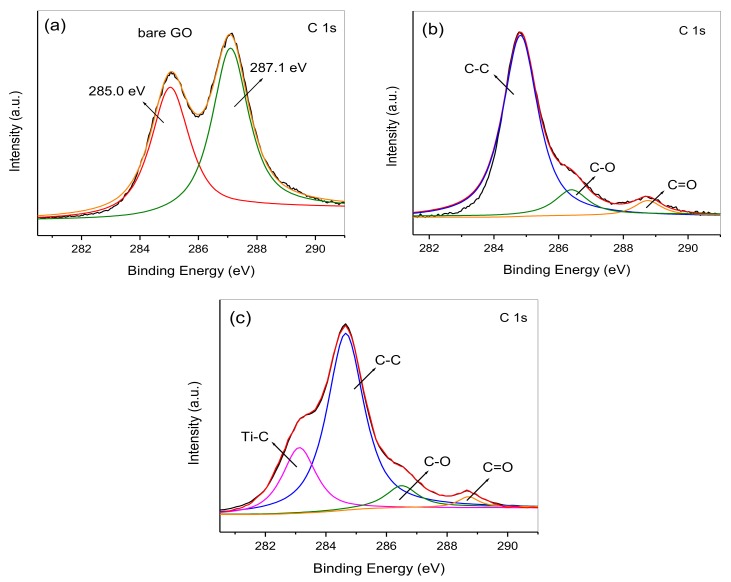
C 1s XPS spectra of (**a**) bare GO, (**b**) HTT/graphene-1.0%-mixed, and (**c**) HTT/graphene-1.0% chemically bonded nanocomposites.

The interactions between HTT and graphene are further studied by XPS. The high-resolution C 1s XPS spectrum of bare GO is shown in Figure 5a. Two characteristic peaks can be observed at 285.0 and 287.1 eV, which correspond to adventitious carbon or sp^2^-hybridized carbon of GO and the oxygen-containing carbonaceous bands (C–OH) [20,39]. These oxygen-containing bands can provide active anchoring sites for HTT growth. The C 1s spectrum of the HTT/graphene-1.0%-mixed sample is deconvoluted into three peaks at binding energies of 284.6, 286.5, and 288.6 eV. The C 1s peaks located at 284.6 and 286.5 eV can be assigned to C–O or C=O groups (Figure 5b). The relative weak peak at 288.6 eV belongs to the O=C–O group. These results indicate that the –OH groups of HTT are likely to interact with the surface –COOH groups of GO via forming O=C–O bonds. Additionally, it can be seen the peak at 287.1 eV, belonging to the C–OH groups of GO, decreases significantly or disappears in the C 1s XPS spectra of HTT/graphene composites. This further demonstrates the successful reduction of GO to graphene through the hydrothermal process. The C 1s spectrum of in-situ synthesized HTT/graphene-1.0% can be deconvoluted into four peaks (Figure 5c). Besides the three peaks at 284.6, 286.5, and 288.6 eV— belonging to the C–C, C–O, and O=C–O groups—another shoulder peak centered at 283.2 eV is observed, which is attributed to forming chemical bonds (Ti–C) between carbon atoms and titanium atoms in the lattice of HTT. This is the first report on the formation of Ti–C bonds in nanocomposites of hydrogen titanate and graphene. The intimate interfacial contacts originated from the chemical bonding would benefit the migration of photogenerated charges and thus improve the photocatalytic activity of HTT.

**Figure 6:**
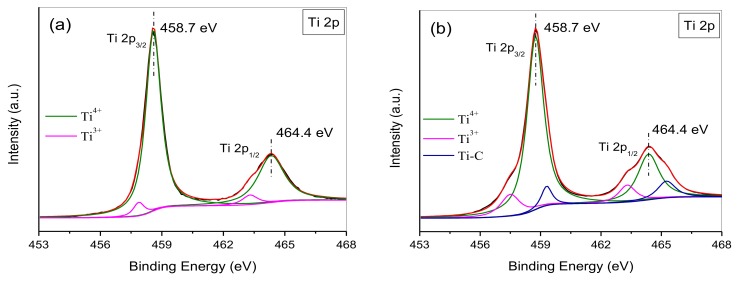
Ti 2p XPS spectra of (**a**) HTT/graphene-1.0%-mixed and (**b**) HTT/graphene-1.0% chemically bonded nanocomposites.

Forming Ti–C bonds is also investigated and identified by analyzing the Ti 2p XPS spectra of the HTT/graphene nanocomposites. As depicted in Figure 6a, in the Ti 2p spectrum of HTT/graphene-1.0%-mixed, two peaks are observed at binding energies of 464.4 and 458.7 eV. These peaks can be attributable to the Ti 2p_1/2_ and Ti 2p_3/2_ spin-orbital splitting photoelectrons of Ti^4+^. Two bands at binding energies of 463.3 and 457.7 eV usually correspond to Ti^3+^ [21,36]. In Figure 6b, besides the above-mentioned four characteristic peaks belonging to Ti^4+^ and Ti^3+^ in HTT, two other weak peaks located at 465.3 and 459.4 eV are found and are likely due to the forming of Ti–C bonds in the HTT/graphene nanohybrids. This suggests that a chemical interaction between HTT and graphene is present in as-synthesized HTT/graphene composites and absent in the mixed samples.

### 3.4. Enhanced Visible Light Catalytic Activities

**Figure 7:**
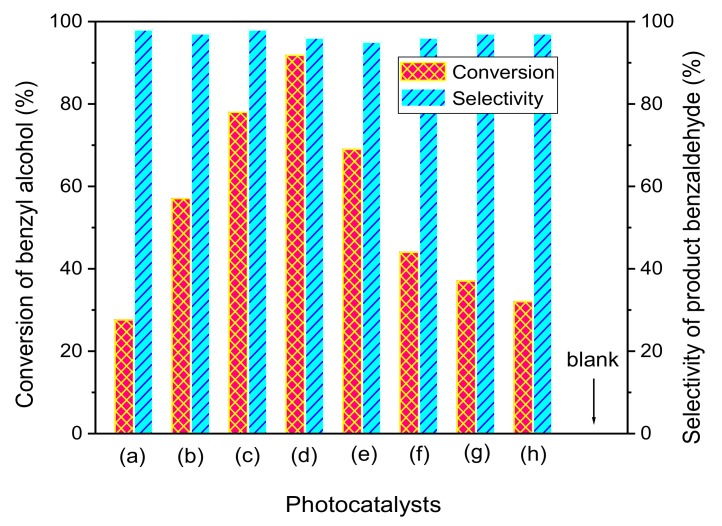
The selective oxidation of benzyl alcohol over HTT/graphene nanocomposites under visible light irradiation: (a) pure HTT, (b) HTT/graphene-0.2%, (c) HTT/graphene-0.4%, (d) HTT/graphene-1.0%, (e) HTT/graphene-2.0%, (f) HTT/graphene-4.0%, (g) P25 TiO_2_/graphene-1.0%, and (h) HTT/graphene-1.0%-mixed. The blank column represents the experimental results in the absence of visible irradiation or photocatalyst.

To investigate the photocatalytic performance of HTT/graphene nanohybrids, the selective oxidation of benzylic alcohols was chosen as a model reaction. No product of aldehyde was obtained without visible radiation or the photocatalyst, indicating that the selective oxidation of benzyl alcohol is conducted through the photocatalytic reaction. Figure 7 shows a comparison of photocatalytic oxidation activity of pure HTT, HTT/graphene-0.2%, HTT/graphene-0.4%, HTT/graphene-1.0%, HTT/graphene-2.0%, HTT/graphene-4.0%, HTT/graphene-1.0%-mixed, and P25 TiO_2_/graphene -1.0%. As depicted in Figure 7, without graphene, pure HTT shows moderate photocatalytic activity and 27.6% of benzyl alcohol was converted into benzaldehyde after 6 h of visible light irradiation. Even when the weight content of graphene is low (0~4.0 wt %), HTT/graphene nanohybrids synthesized using the in-situ compositing approach exhibit remarkably enhanced photocatalytic activities, and the prepared photocatalysts possess high selectivity (>95%) for the conversion of benzyl alcohol to benzaldehyde. The photo-oxidation activity of HTT/graphene increases with graphene amount from 0.2 to 1.0 wt %. The optimal conversion yield of benzyl alcohol, obtained in the sample HTT/graphene-1.0%, is 91.8%, which exceeds by 3.3 times that of pure HTT. To our knowledge, this is the first report demonstrating that hydrogen titanate/graphene composites serve as efficient visible light catalysts toward the partial oxidation of benzylic alcohols. When the content of graphene is above 1.0%, further increasing the graphene content results in a reduction of photo-activity. For instance, the conversion yield of benzyl alcohol over HTT/graphene-4.0% has a sharp decline. This may be due to the increased turbidity and light scattering of HTT/graphene-4.0%. Additionally, high loading of graphene shields HTT from absorbing light irradiation, which leads to an obvious reduction of illumination passing through the reaction suspension. The results are analogous to the previous studies of TiO_2_/graphene nanohybrids for contaminant degradation and hydrogen production [19,20].

For comparison, the selective conversion of benzyl alcohol in P25 TiO_2_/graphene-1.0% and HTT/graphene-1.0%-mixed have also been tested and analyzed under the same experimental conditions. The sample of P25 TiO_2_/graphene-1.0% shows dramatically lower activity (37.0% of alcohol is oxidized) than the HTT/graphene-1.0% composites, suggesting that more direct contact and the resulting strong interactions between HTT and graphene sheets are critical for the enhancement of photocatalytic activity because of improved electron/hole separation and migration. The physical mixed sample (HTT/graphene-1.0%-mixed) shows a significantly lower conversion yield than that of HTT/graphene-1.0% synthesized via a one-step hydrothermal process. This suggests that simple mechanical-mixing with graphene has a small effect on improving the photocatalytic activity of HTT, and is due to the absence of a chemical-bonding interface between HTT and graphene. This also indicates that the interaction state between HTT and graphene has crucial influence on photocatalytic performance of HTT/graphene nanohybrids.

**Figure 8:**
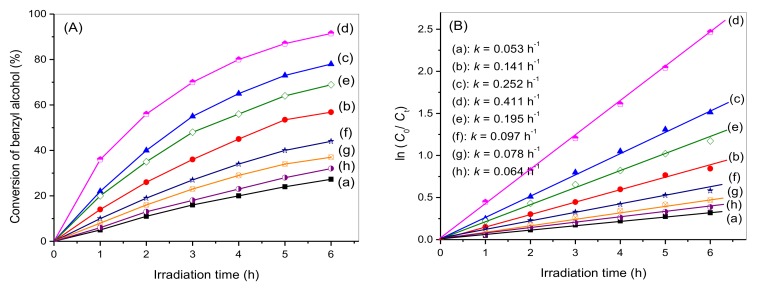
(**A**) Time involved photocatalytic conversion of benzyl alcohol over as-synthesized HTT/graphene nanocomposites: (a) pure HTT, (b) HTT/graphene-0.2%, (c) HTT/graphene-0.4%, (d) HTT/graphene-1.0%, (e) HTT/graphene-2.0%, (f) HTT/graphene -4.0%, (g) P25 TiO_2_/graphene-1.0%, and (h) HTT/graphene-1.0%-mixed; (**B**) The corresponding first-order kinetics plots over these photocatalysts.

Additionally, the reaction kinetics of benzyl alcohol oxidation were investigated and the corresponding results are shown in Figure 8. As the reaction time is prolonged, slight decreases in the conversion yields of benzyl alcohol occur. Derived from the experimental data in Figure 8A, the kinetics of benzyl alcohol oxidation are shown in Figure 8B. A linear correlation between ln(C_0_/C_t_) (C_0_ and C_t_ are the concentrations of benzyl alcohol at the initial time and at specific time intervals) and the reaction time is displayed, indicating the oxidation is pseudo-first order. The pseudo-first-order rate constant (*k*) of the HTT/graphene photocatalysts can be obtained from the slope. The rate constant (*k*) of benzyl alcohol oxidation over the optimal photocatalyst, HTT/graphene-1.0%, is 0.411 h^−1^, which is 5.3 and 6.4 times higher than those of the P25 TiO_2_/graphene-1.0% and HTT/graphene-1.0% -mixed samples.

To investigate the general applicability of HTT/graphene composites, the oxidation of various benzylic alcohols over the optimal HTT/graphene-1.0% were examined and the corresponding results are displayed in Table 2. It can be seen from Table 2 that the selective conversion of various benzylic alcohol derivatives proceeds, and the desirable aldehyde products are obtained in high selectivity (entries 1–5) under visible radiation. Additionally, it can be noted that the electron-donating substituents (CH_3_– and CH_3_O–) facilitate the formation of aldehyde products; for example, 97.6% of 4-methoxybenzyl alcohol can be converted in 4.5 h with 96% selectivity. Whereas, the electron-withdrawing substituents (such as F– and Cl–) retard the conversion of alcohols. This suggests that the existence of an electron-donating group at the *p*-position of benzyl alcohol benefits the selective conversion over an HTT/graphene photocatalyst.

### 3.5. Stability of Composite Photocatalysts

**Figure 9:**
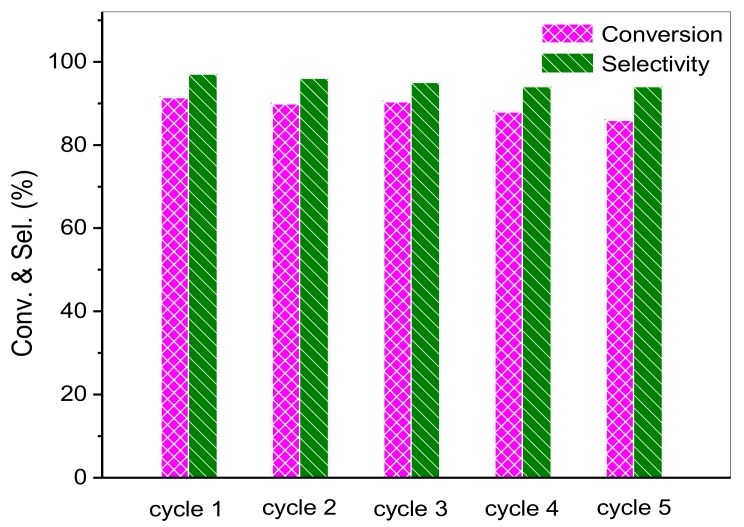
Recycled photoactivity test for five operational runs over the optimal HTT/graphene-1.0%, on selective oxidation of benzyl alcohol.

The stability or recyclability of catalysts is another important practical issue. In order to study the stability and durability of HTT/graphene composites, cycle experiments are performed. After the catalytic reactions, HTT/graphene-1.0% photocatalysts are separated and recovered by washing, and are reused. As depicted in Figure 9, there is only a small loss of activity and no obvious change of selectivity after five consecutive runs for partial photo-oxidation of benzyl alcohol. These results indicate that HTT/graphene is an efficient and stable photocatalyst for selective conversion of benzyl alcohol under the applied experimental conditions.

### 3.6. Visible Light Absorption Generated by Benzyl Alcohol Surface Adsorption

**Figure 10:**
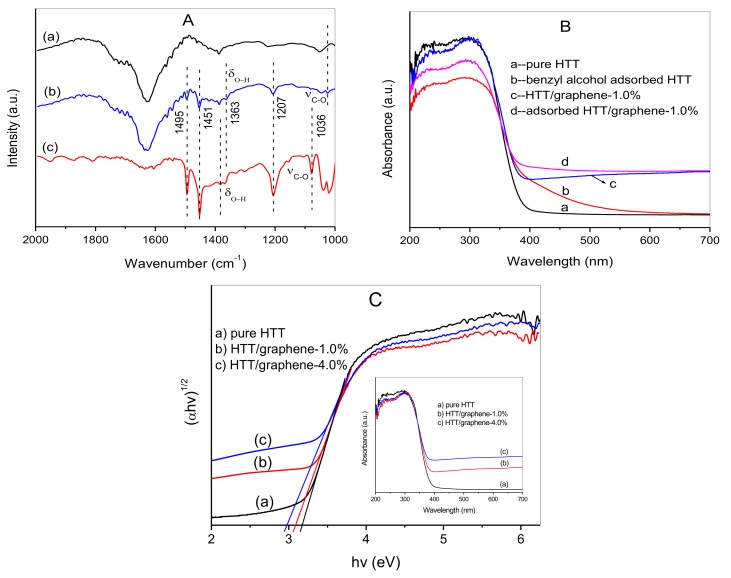
(**A**) FTIR spectra of (a) HTT/graphene-1.0%, (b) benzyl alcohol adsorbed HTT /graphene-1.0% and (c) free benzyl alcohol; (**B**) UV-vis DRS of pure HTT, benzyl alcohol adsorbed HTT, HTT/graphene-1.0%, and benzyl alcohol adsorbed HTT/graphene-1.0%; (**C**) Plot of transformed Kubelka–Munk function versus the energy of light (Inset: the corresponding UV-vis DRS).

Surface adsorption generally plays a critical role toward photocatalysis-driven selective oxidation of alcohols. The surface adsorption of benzyl alcohol on HTT/graphene composites is first investigated using FTIR spectra (Figure 10A). In comparison with free benzyl alcohol molecules, the characteristic peaks assigned to C–O and O–H groups shift to lower wavenumbers (at 1036 and 1363 cm^−1^) after being adsorbed over HTT/graphene-1.0%. On the other hand, the characteristic peaks belonging to skeletal vibrations of the phenyl ring (at 1495 and 1451 cm^−1^) and the C–C stretching vibration (at 1207 cm^−1^) show no changes [26,28]. These results imply that benzyl alcohol is adsorbed on the surface of HTT/graphene and the –CH_2_OH groups of the alcohol molecules interact with the unsaturated Ti sites of HTT, forming alcoholate species –CH_2_–O(H)–Ti.

The formation of surface complexes –CH_2_–O(H)–Ti can produce visible absorption, which provides a prior condition for the selective conversion of benzylic alcohol to product aldehyde. This has been previously shown in research on photocatalytic reactions over TiO_2_ or Nb_2_O_5_ [40,41]. Based on the literature, surface-unsaturated metals (such as Ti or Nb) can serve as Lewis acid sites and the alcohol molecules are adsorbed via Lewis acid-base interactions. The visible light absorption can be attributed to the ligand-to-metal charge transfer (LMCT) of in-situ formed surface complexes. The visible light absorption caused by the surface adsorption of benzyl alcohol over pure HTT and HTT/graphene-1.0% is indicated in Figure 10B. As presented in Figure 10B, the pure HTT sample shows no visible light response, whereas the benzyl alcohol adsorbed HTT exhibits obvious visible light absorption. This absorption can be ascribed to the formation of surface complexes between adsorbed benzyl alcohol and the unsaturated surface Ti atoms of HTT. It can be concluded from Figure 10B that the incorporation of graphene results in continuous absorption bands in visible light range for HTT/graphene composites, which is analogous to results on TiO_2_/graphene nanohybrids [42]. Although the HTT/graphene nanocomposites extend the wide background absorption in visible light region, additional visible light absorption can be observed for benzyl alcohol adsorbed composites. This demonstrates that the introduction of graphene has no influence on the formation of surface complexes.

The band gap energy of the photocatalyst is an important factor affecting the catalytic performance, especially for a visible light photocatalytic system. The band gap energies of pure HTT, HTT/graphene-1.0%, and HTT/graphene-4.0% are estimated through a Tauc plot of the modified Kubelka–Munk function with linear extrapolations (Figure 10C). The calculated band gap energies are 3.16, 3.09, 2.98 eV for pure HTT, HTT/graphene-1.0%, and HTT/graphene-4.0%, respectively. The reduction in band gap energy can be ascribed to chemical bonding between HTT and the graphene support. As shown in the inset of Figure 10C, a slight red-shift in the absorption edge of the HTT/graphene composites is observed. For instance, the absorption edge of HTT/graphene-1.0% is about 401 nm, which implies that HTT/graphene-1.0% can hardly absorb visible light. That is, the red-shift originating from the chemical bonding between HTT and graphene is insufficient to bring forth excellent catalytic activity for the selective oxidation of benzylic alcohols. Hence, the visible light oxidation of benzylic alcohols over HTT/graphene nanocomposites is mainly driven by the formation of surface complexes.

### 3.7. Improved Charge Transfer via the Chemically Bonded Interface between HTT and Graphene

**Figure 11:**
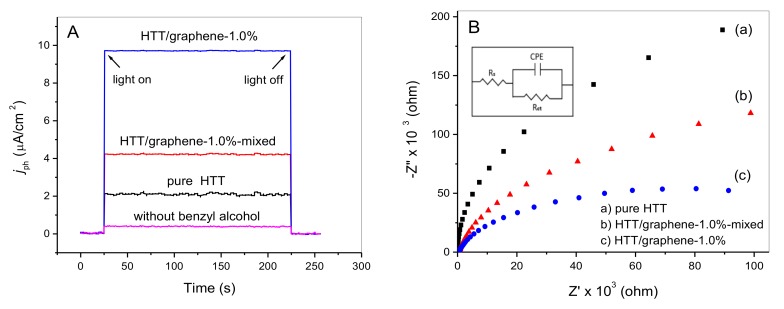
(**A**) Photocurrent curves of pure HTT, HTT/graphene-1.0%-mixed, and HTT/graphene-1.0% electrodes under visible light irradiation; the violet curve represents the photocurrent response over HTT/graphene-1.0% electrode without benzyl alcohol in the electrolyte solution. (**B**) Electrochemical impedance spectra (EIS) Nyquist plots of the pure HTT, HTT/graphene-1.0%-mixed, and HTT/graphene-1.0% samples under light irradiation with a Xe lamp. Inset: Electrical equivalent circuit supposed for fitting of impedance spectra. R_S_, CPE, and R_ct_ represent the electrolyte resistance, space charge capacitance, and the charge transfer resistance, respectively.

To investigate visible light induced charge transfer during the photo-oxidation of benzylic alcohols over HTT/graphene catalysts, the transient photocurrent responses were recorded over working electrodes made of pure HTT, HTT/graphene-1.0%-mixed and in-situ compositing HTT/graphene-1.0%. Figure 11A presents the *I*-*t* curves of the above-mentioned three working electrodes in a benzyl alcohol containing electrolyte solution under visible light illumination. The photocurrent is primarily determined by the charge transfer from the adsorbed benzyl alcohols to the HTT conduction band and the subsequent graphene surface. The photocurrent density of the HTT/graphene-1.0% electrode is enhanced ~4.6 times that of pure HTT, indicating the separation efficiency of photoinduced charges can be improved by the interfacial electronic interactions between HTT and graphene. Moreover, the photocurrent density of the in-situ composited HTT/graphene-1.0% is enhanced ~2.3 times that of the mechanical-mixed hybrid (HTT/graphene-1.0%-mixed), which is primarily ascribed to the chemical interactions between HTT and the graphene sheets. Additionally, for the HTT/graphene-1.0% electrode, the photocurrent density in the presence of benzyl alcohol is predominantly higher than that in its absence. This result implies the generation of anodic photocurrent is closely related to the surface adsorption of benzyl alcohol upon the HTT/graphene-1.0% electrode. The photocurrent experiments offer direct evidence for electron migration from the adsorbed benzyl alcohol to the conduction band of HTT and subsequently to graphene surface under visible light.

Based on the experimental data in Figure 11A and Table 1, the photocurrent results are in accordance with the *S*_BET_ normalized rate constants of benzyl alcohol oxidation. This suggests that visible light induced charge transfer plays a pivotal role in the photocatalytic conversion of benzylic alcohol. For graphene-based photocatalytic systems, the enhanced charge separation efficiency is dominated by the high spatial charge transportation properties of graphene [43,44]. The extent of interaction between graphene and semiconductor catalysts determines the electron migration, further influencing the photocatalytic activity [19,20,21,22]. The formation of a chemically bonded interface between catalyst nanoparticles and graphene can improve the photocatalytic performance of semiconductor catalysts [19,20,21,22]. Based on the above characterization results (Figure 5, Figure 6 and Figure 11A) and photocatalytic experimental data (Figure 8 and Table 1), an efficient interfacial charge transfer based on a chemical-bonding effect is proposed to clarify the promoted catalytic activity of HTT/graphene nanocomposites, especially compared to mechanically mixed samples. The improved charge separation caused by the interaction extent between HTT and graphene sheets can be further investigated by EIS.

Figure 11B compares the EIS Nyquist curves of the in situ-synthesized and mechanically mixed HTT/graphene composites under irradiation by a xenon lamp. Figure 11B shows only one semicircle on the EIS plan, which suggests that charge migration is occurring, and the supposed equivalent circuit is given in the inset of Figure 11B. The charge transfer resistances of the pure HTT, HTT/graphene-1.0%-mixed, and HTT/graphene-1.0% samples are 4.9 × 10^5^, 2.2 × 10^5,^ and 1.0 × 10^5^ ohm, determined by fitting with the equivalent circuit. This demonstrates that the introduction of graphene facilitates the charge transfer of HTT. Furthermore, the arc radius of the EIS Nyquist curve for HTT/graphene-1.0% is smaller than that of both the physically mixed (HTT/graphene-1.0%-mixed) and pure HTT samples. Generally, the arc radius of an EIS spectrum reflects the resistance of the interface layer at the surface of the electrodes [37,45]. The smaller semicircle size represents more efficient separation of electrons/holes and more rapid interfacial charge transfer [37,45]. These results clearly reveal that the chemical bonding of HTT and graphene can efficiently promote the migration and separation of photogenerated charges, contributing to improve the catalytic performance for selective oxidation of benzylic alcohols.

### 3.8. Radical Trapping Experiments

**Figure 12:**
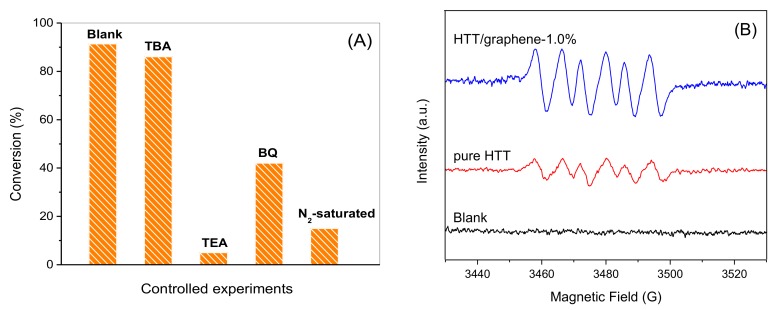
(**A**) Control experiments of photocatalytic selective oxidation of benzyl alcohol in the presence of various scavengers or in the absence of O_2_ over HTT/graphene-1.0% under visible light irradiation for 6 h. (**B**) Electron spin resonance (ESR) spectra of O_2_^·^ˉ trapped by DMPO over pure HTT and HTT/graphene-1.0% suspensions in methanol solution, blank represents both the sample containing DMPO alone under light illumination and the sample containing DMPO and catalysts in dark.

The involved catalytic mechanism of alcohol photo-oxidation on HTT/graphene composites is further studied. Control experiments on radical trapping were carried out and the obtained results are illustrated in Figure 12A. When adding *tert*-butyl alcohol (TBA) to the reaction solution [46], the conversion of benzyl alcohol is almost unchanged, indicating ·OH has little effect on the selective conversion of benzyl alcohol. Removing O_2_ by sparging with N_2_ results in a low conversion of benzyl alcohol (ca. 15%), indicating that O_2_ plays a critical role in photocatalytic oxidation of benzyl alcohol. When triethanolamine (TEA, a scavenger of photohole h^+^) is put into the reaction system, the oxidation of benzyl alcohol is remarkably inhibited, implying that partial oxidation of benzyl alcohol is primarily driven by photo-holes. Additionally, the introduction of benzoquinone (BQ) results in an obvious decrease in the conversion of benzyl alcohol, suggesting that O_2_^·^ˉ is also involved during the photo-oxidation of benzyl alcohol. To confirm the formation of O_2_^·^ˉ, active radical species generated over HTT/graphene-1.0% are detected by ESR using DMPO as the spin-trapping agent. As depicted in Figure 12B, after irradiation of the HTT suspension for 5 min, the characteristic spectrum of the adduct DMPO/·OOH formed between DMPO and superoxide radicals can be clearly observed. In the presence of HTT/graphene-1.0%, the signal intensity of DMPO/·OOH increased. These results indicate that superoxide radicals can be formed upon pure HTT or HTT/graphene under light illumination, and that introduction of graphene significantly enhances the formation of O_2_^·^ˉ.

### 3.9. Mechanism of Improved Visible Light Catalytic Activity

**Figure 13:**
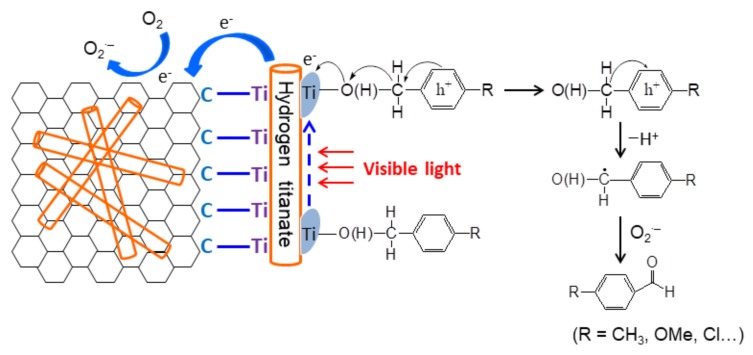
Schematic illustrations for the efficient interfacial charge transfer effect over HTT/graphene composites toward the selective oxidation of benzylic alcohols under visible light irradiation.

According to these results, a possible mechanism for the improved visible light catalytic activity over as-prepared HTT/graphene composites is proposed and depicted in Figure 13. Reactant benzylic alcohol is first adsorbed on unsaturated Ti site of HTT and generates the surface complex. A visible light induced LMCT process occurs, which produces an electron and a hole upon the in-situ formed surface complex. The photogenerated electron transfers into the conduction band of neighbor HTT and subsequently the surface of the graphene sheet via the in-situ formed Ti–C bonds, which can be trapped by adsorbed O_2_ to produce a superoxide anion radical. Meanwhile, the adsorbed benzylic alcohol transforms into an intermediate with a positive charge. Subsequently, the photogenerated hole drives the α-deprotonation of the intermediate and gives rise to a neutral carbon radical. Then the reaction between the substrate radical and superoxide species yields the product aldehyde. Compared to the HTT/graphene-mixed samples, the in-situ composited HTT/graphene catalysts possess significantly enhanced photocatalytic activity for the selective oxidation of benzylic alcohols under visible light illumination, as indicated in Figure 8 and Table 1. It can be mainly ascribed to the chemical-bonding effect and the resulting intimate interactions between HTT and graphene sheets, which facilitate the interfacial electron transfer from adsorbed alcohols to HTT and finally to graphene. The close interfacial contacts between graphene and HTT are not being able to form in the mechanical mixed composites. In brief, the improved oxidation efficiency of as-synthesized HTT/graphene composites is primarily ascribed to the interfacial charge transfer effect, which inhibits the recombination of photoinduced electron-hole pairs and thus enhances the photoactivity of alcohol oxidation.

## 4. Conclusions

In summary, HTT/graphene nanohybrids with superior photocatalytic activity were fabricated through a facile hydrothermal process. Nanotubular HTT are densely supported upon the surface of graphene with close interfacial contacts. Introducing graphene can markedly improve the catalytic performance for the selective conversion of benzylic alcohols under visible illumination and the contents of graphene have significant effects on the catalytic activity of HTT. The optimal content of graphene is 1.0 wt % and the corresponding normalized rate constant k of benzyl alcohol oxidation is 1.71 × 10^−3^ g/m^2^·h, which exceeds that of pure HTT by more than seven times. Further characterization results indicate that HTT are chemically bonded with graphene surfaces via Ti–C bonds, which is supported by XPS analysis. More importantly, the chemical-bonded nanocomposite exhibits a remarkable enhancement of photocatalytic activity, which is 5.2 times that of a mechanical-mixed sample. Based on the results of transient photocurrent and EIS, this enhancement can be ascribed to the interfacial charge transfer effect, which provides favorable spatial conditions for electrons migration from in-situ generated surface complexes to neighboring HTT and finally to graphene. This effect inhibits the recombination of electrons and holes, and thus yields superior photocatalytic performance. Our study on the efficient interfacial charge transfer via a chemically bonded interface provides new insights for guiding the design of novel photocatalytic reaction systems, particularly for solar-light driven selective organic synthesis.

## Figures and Tables

**Table 1:** Brunauer–Emmett–Teller (BET) surface area of as-prepared HTT/graphene composites and *S*_BET_ normalized rate constants *k* for the selective oxidation of benzyl alcohol.

Catalyst	BET Surface Area/m^2^·g^−1^	Conv./%	Rate Constant *k*/h^−1^	*S*_BET_ Normalized Rate Constants *k*
pure HTT	225.1	27.6	0.053	0.24 × 10^−3^
HTT/graphene-0.2%	228.9	57.1	0.141	0.61 × 10^−3^
HTT/graphene-0.4%	234.4	78.0	0.252	1.08 × 10^−3^
HTT/graphene-1.0%	240.5	91.8	0.411	1.71 × 10^−3^
HTT/graphene-2.0%	244.7	69.2	0.195	0.80 × 10^−3^
HTT/graphene-4.0%	248.0	43.8	0.097	0.39 × 10^−3^
HTT/graphene-1.0%-mixed	233.6	32.3	0.078	0.33 × 10^−3^

Reaction conditions: catalyst 50 mg, benzylic alcohol 0.25 mmol, acetonitrile 5 mL, reaction temperature 298 K, visible light irradiation 6 h.

**Table 2:** Photocatalytic oxidation of various benzylic alcohols over HTT/graphene-1.0%.

Entry	Substrate	Product	Time (h)	Conv. (%)	Sel. (%)
1	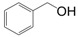	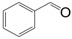	6	91.8	96
2	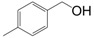	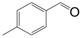	5	92.5	97
3	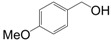	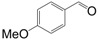	4.5	97.6	96
4	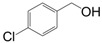	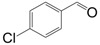	7	89.2	97
5	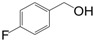	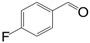	9	84.9	98

Reaction conditions: catalyst 50 mg, benzylic alcohol 0.25 mmol, acetonitrile 5 mL, reaction temperature 298 K, visible light irradiation (λ > 400 nm).

## References

[1] Chen C.C., Ma W.H., Zhao J.C. (2010). Semiconductor-mediated photodegradation of pollutants under visible-light irradiation. Chem. Soc. Rev..

[2] Kamat P.V. (2007). Meeting the clean energy demand: Nanostructure architectures for solar energy conversion. J. Phys. Chem. C.

[3] Lang X.J., Ma W.H., Chen C.C., Ji H.W., Zhao J.C. (2014). Selective aerobic oxidation mediated by TiO_2_ photocatalysis. Acc. Chem. Res..

[4] Humayun M., Li Z.J., Sun L.Q., Zhang X.L., Raziq F., Zada A., Qu Y., Jing L.Q. (2016). Coupling of nanocrystalline anatase TiO_2_ to aporous nanosized LaFeO_3_ for efficient visible-light photocatalytic degradation of pollutants. Nanomaterials.

[5] Yurdakal S., Palmisano G., Loddo V., Augugliaro V., Palmisano L. (2008). Nanostructured rutile TiO_2_ for selective photocatalytic oxidation of aromatic alcohols to aldehydes in water. J. Am. Chem. Soc..

[6] Yang D.J., Liu H.W., Zheng Z.F., Yuan Y., Zhao J.C., Waclawik E.R., Ke X.B., Zhu H.Y. (2009). An efficient photocatalyst structure: TiO_2_(B) nanofibers with a shell of anatase nanocrystals. J. Am. Chem. Soc..

[7] Liu Y.X., Wang Z.L., Wang W.D., Huang W.X. (2014). Engineering highly active TiO_2_ photocatalysts via the surface-phase junction strategy employing a titanate nanotube precursor. J. Catal..

[8] Zheng Z.K., Huang B.B., Lu J.B., Wang Z.Y., Qin X.Y., Zhang X.Y., Dai Y., Whangbo M.H. (2012). Hydrogenated titania: Synergy of surface modification and morphology improvement for enhanced photocatalytic activity. Chem. Commun..

[9] Chatterjee S., Bhattacharyya K., Ayyub P., Tyagi A.K. (2010). Photocatalytic properties of one-dimensional nanostructured titanates. J. Phys. Chem. C.

[10] Yu J.G., Xiang Q.J., Zhou M.H. (2009). Preparation, characterization and visible-light-driven photocatalytic activity of Fe-doped titania nanorods and first-principles study for electronic structures. Appl. Catal. B-Environ..

[11] Zheng Z.F., Liu H.W., Ye J.P., Zhao J.C., Waclawika E.R., Zhu H.Y. (2010). Structure and contribution to photocatalytic activity of the interfaces in nanofibers with mixed anatase and TiO_2_(B) phases. J. Mol. Catal. A-Chem..

[12] Turki A., Kochkar H., Guillard C., Berhault G., Ghorbel A. (2013). Effect of Na content and thermal treatment of titanate nanotubes on the photocatalytic degradation of formic acid. Appl. Catal. B-Environ..

[13] Tang Z.R., Li F., Zhang Y.H., Fu X.Z., Xu Y.-J. (2011). Composites of titanate nanotube and carbon nanotube as photocatalyst with high mineralization ratio for gas-phase degradation of volatile aromatic pollutant. J. Phys. Chem. C.

[14] Kim I.Y., Lee J.M., Kim T.W., Kim H.N., Kim H., Choi W., Hwang S.-J. (2012). A strong electronic coupling between graphene nanosheets and layered titanate nanoplates: A soft-chemical route to highly porous nanocomposites with improved photocatalytic activity. Small.

[15] Liu G.G., Han K., Ye H.Q., Zhu C.Y., Gao Y.P., Liu Y., Zhou Y.H. (2017). Graphene oxide/triethanolamine modified titanate nanowires as photocatalytic membrane for water treatment. Chem. Eng. J..

[16] Kim S., Han K.I., Lee I.G., Park W.K., Yoon Y., Yoo C.S., Yang W.S., Hwang W.S. (2016). A gallium oxide-graphene oxide hybrid composite for enhanced photocatalytic reaction. Nanomaterials.

[17] Chen Y., Sun H.Q., Peng W.C. (2017). 2D transition metal dichalcogenides and graphene-based ternary composites for photocatalytic hydrogen evolution and pollutants degradation. Nanomaterials.

[18] Zhang N., Yang M.-Q., Tang Z.-R., Xu Y.-J. (2013). CdS-graphene nanocomposites as visible light photocatalyst for redox reactions in water: A green route for selective transformation and environmental remediation. J. Catal..

[19] Zhang H., Lv X.J., Li Y.M., Wang Y., Li J.H. (2010). P25-graphene composite as a high performance photocatalyst. ACS Nano.

[20] Sun Z.H., Guo J.J., Zhu S.M., Mao L., Ma J., Zhang D. (2014). A high-performance Bi_2_WO_6_-graphene photocatalyst for visible light-induced H_2_ and O_2_ generation. Nanoscale.

[21] Gao F.D., Zeng D.W., Huang Q.W., Tian S.Q., Xie C.S. (2012). Chemically bonded graphene/BiOCl nanocomposites as high-performance photocatalysts. Phys. Chem. Chem. Phys..

[22] Huang Q.W., Tian S.Q., Zeng D.W., Wang X.X., Song W.L., Li Y.Y., Xiao W., Xie C.S. (2013). Enhanced photocatalytic activity of chemically bonded TiO_2_/graphene composites based on the effective interfacial charge transfer through the C-Ti bond. ACS Catal..

[23] Palmisano G., Augugliaro V., Pagliaro M., Palmisano L. (2007). Photocatalysis: A promising route for 21st century organic chemistry. Chem. Commun..

[24] Zhang M., Wang Q., Chen C., Zang L., Ma W., Zhao J. (2009). Oxygen atom transfer in the photocatalytic oxidation of alcohols by TiO_2_: Oxygen isotope studies. Angew. Chem. Int. Ed..

[25] Tanaka A., Hashimoto K.J., Kominami H. (2012). Preparation of Au/CeO_2_ exhibiting strong surface plasmon resonance effective for selective or chemoselective oxidation of alcohols to aldehydes or ketones in aqueous suspensions under irradiation by green light. J. Am. Chem. Soc..

[26] Liang S.J., Wen L.R., Lin S., Bi J.H., Feng P.Y., Fu X.Z., Wu L. (2014). Monolayer HNb_3_O_8_ for selective photocatalytic oxidation of benzylic alcohols with visible light response. Angew. Chem. Int. Ed..

[27] Higashimoto S., Kitao N., Yoshida N., Sakura T., Azuma M., Ohue H., Sakata Y. (2009). Selective photocatalytic oxidation of benzyl alcohol and its derivatives into corresponding aldehydes by molecular oxygen on titanium dioxide under visible light irradiation. J. Catal..

[28] Yang J., Shen X.X., Wei J.P., Zhang L.N., Zhao D., Yao B.H. (2016). Selective oxidation of alcohols on hydrogen titanate nanotubes under visible light irradiation: Relationship between nanostructure and catalytic activity. Catal. Sci. Technol..

[29] Yu L.H., Lin Y.M., Li D.Z. (2017). Visible-light-induced aerobic oxidation of alcohols in a green catalytic system of carbonate-like species doped TiO_2_. Appl. Catal. B-Environ..

[30] Andryushina N.S., Stroyuk O.L. (2014). Influence of colloidal graphene oxide on photocatalytic activity of nanocrystalline TiO_2_ in gas-phase ethanol and benzene oxidation. Appl. Catal. B-Environ..

[31] Yang X., Zhao H., Feng J.F., Chen Y.M., Gao S.Y., Cao R. (2017). Visible-light-driven selective oxidation of alcohols using a dye-sensitized TiO_2_-polyoxometalate catalyst. J. Catal..

[32] Zhai Q.Q., Bo T., Hu G.X. (2011). High photoactive and visible-light responsive graphene/titanate nanotubes photocatalysts: Preparation and characterization. J. Hazard. Mater..

[33] Dang H.F., Dong X.F., Dong Y.C., Huang J.S. (2013). Facile and green synthesis of titanate nanotube/graphene nanocomposites for photocatalytic H_2_ generation from water. Int. J. Hydrog. Energy.

[34] Zou J.P., Ma J., Huang Q., Luo S.L., Yu J., Luo X.B., Dai W.L., Sun J., Guo G.C., Au C.T. (2014). Graphene oxide as structure-directing and morphology-controlling agent for the syntheses of heterostructured graphene-Bi_2_MoO_6_/Bi_3.64_Mo_0.36_O_6.55_ composites with high photocatalytic activity. Appl. Catal. B-Environ..

[35] Sun X., Li Y. (2003). Synthesis and characterization of ion-exchangeable titanate nanotubes. Chem.-Eur. J..

[36] Perera S.D., Mariano R.G., Vu K., Nour N., Seitz O., Chabal Y., Balkus K.J. (2012). Hydrothermal synthesis of graphene-TiO_2_ nanotube composites with enhanced photocatalytic activity. ACS Catal..

[37] Yang J., Wang X.H., Zhao X.L., Dai J., Mo S.R. (2015). Synthesis of uniform Bi_2_WO_6_-reduced graphene oxide nanocomposites with significantly enhanced photocatalytic reduction activity. J. Phys. Chem. C.

[38] Manna A.K., Pati S.K. (2009). Tuning the electronic structure of graphene by molecular charge transfer: A computational study. Chem.-Asian J..

[39] Akhavan O., Ghaderi E. (2009). Photocatalytic reduction of graphene oxide nanosheets on TiO_2_ thin Film for photoinactivation of bacteria in solar light irradiation. J. Phys. Chem. C.

[40] Shishido T., Teramura K., Tanaka T. (2011). A unique photo-activation mechanism by “in situ doping” for photo-assisted selective NO reduction with ammonia over TiO_2_ and photooxidation of alcohols over Nb_2_O_5_. Catal. Sci. Technol..

[41] Lang X., Ma W., Zhao Y., Chen C., Ji H., Zhao J. (2012). Visible-light-induced selective photocatalytic aerobic oxidation of amines into imines on TiO_2_. Chem.-Eur. J..

[42] Yang M.Q., Zhang N., Xu Y.J. (2013). Synthesis of fullerene-, carbon nanotube-, and graphene-TiO_2_ nanocomposite photocatalysts for selective oxidation: A comparative study. ACS Appl. Mater. Inter..

[43] Chen D., Zhang H., Liu Y., Li J.H. (2013). Graphene and its derivatives for the development of solar cells, photoelectrochemical, and photocatalytic applications. Energy Environ. Sci..

[44] Xiang Q.J., Yu J.G. (2013). Graphene-based photocatalysts for hydrogen generation. J. Phys. Chem. Lett..

[45] Xiao F.X., Wang F.C., Fu X.Z., Zheng Y. (2012). A green and facile self-assembly preparation of gold nanoparticles/ZnO nanocomposite for photocatalytic and photoelectrochemical applications. J. Mater. Chem..

[46] Xiao X.Y., Jiang J., Zhang L.Z. (2013). Selective oxidation of benzyl alcohol into benzaldehyde over semiconductors under visible light: The case of Bi_12_O_17_Cl_2_ nanobelts. Appl. Catal. B-Environ..

